# Genetic polymorphisms and response to 5-fluorouracil, doxorubicin and cyclophosphamide chemotherapy in breast cancer patients

**DOI:** 10.18632/oncotarget.11053

**Published:** 2016-08-04

**Authors:** Karolina Tecza, Jolanta Pamula-Pilat, Joanna Lanuszewska, Ewa Grzybowska

**Affiliations:** ^1^ Center for Translational Research and Molecular Biology of Cancer, Maria Sklodowska-Curie Memorial Cancer Center and Institute of Oncology, Gliwice Branch, Warsaw, Poland

**Keywords:** breast cancer, FAC, chemotherapy, polymorphism, treatment response

## Abstract

Clinical resistance to chemotherapy is one of the major problems in breast cancer treatment. In this study we analyzed possible impact of 22 polymorphic variants on the treatment response in 324 breast cancer patients. Selected genes were involved in FAC chemotherapy drugs transport (*ABCB1*, *ABCC2*, *ABCG2*, *SLC22A16*), metabolism (*CYP1B1*, *CYP2C19*, *GSTT1*, *GSTM1*, *GSTP1*, *TYMS*, *MTHFR*, *DPYD*), drug-induced damage repair (*ERCC1*, *ERCC2*, *XRCC1*) and involved in regulation of DNA damage response and cell cycle control (*ATM*, *TP53*).

Apart from preexisting metastases three polymorphic variants were independent prognostic high risk factors of lack of response to FAC chemotherapy. Our results showed that the response to treatment depended of the variability in genes engaged in drugs’ transport (*ABCC2* c.-24C>T, *ABCB1* p.Ser893Ala/Thr) and in DNA repair machinery (*ERCC2* p.Lys751Gln). Furthermore, the growing number of high-risk genotypes was reflected in gradual increase in risk of the non-responsiveness to treatment- from OR 2.68 for presence of two genotypes to OR 9.93 for carriers of all three negative genotypes in the group of all patients. Similar gene-dosage effect was observed in the subgroup of TNBCs. Also, TFFS significantly shortened with the increasing number of high-risk genotypes, with median of 54.4 months for carriers of one variant, to 51.5 and 34.9 months for the carriers of two and three genotypes, respectively.

Our results demonstrate that results of cancer treatment are the effect of many clinical and genetic factors. It seems that multifactorial polymorphic models could be a potentially useful tool in personalization of cancer therapies. The novelty in our model is the over representation of triple negative breast cancer (TNBC) patients among the carriers of all unfavorable polymorphic variants. This finding contributes to the elucidation of the mechanisms of drug resistance in this subgroup of breast cancer patients.

## INTRODUCTION

Clinical resistance to breast cancer chemotherapy is observed as incidences of disease progression, local recurrence, primary and secondary tumors at different locations, and cancer-related mortality. The primary cause of drug resistance is linked to genetic instability of tumor cells, which leads to accumulation of mutations and chromosomal aberrations. These changes often alter cells’ adhesion and dysregulate apoptotic pathways leading to highly aggressive and immortal phenotype. Apart from tumor-related factors, chemotherapy resistance is associated with patient's body ability to metabolize and remove drugs from the body. The factors that can influence therapeutic potential of a drug are: its reduced transport into tumor cells, overexpression of efflux transporters and modified DNA repair systems which remove drug-induced damage [1, 2].

Chemotherapy consisting of 5-fluorouracil (5-FU), doxorubicin and cyclophosphamide (FAC regime) is commonly used in neo-adjuvant or adjuvant treatment of breast cancer. Resistance to antimetabolite 5-FU may be conferred by alterations in enzymes involved in fluoropyrimidine metabolism, particularly enzymes associated with the conversion of 5-FU to the thymidylate synthase inhibitor FdUMP which is one of the 5-FU active metabolites. Furthermore, changes in the thymidylate synthase level or its affinity for FdUMP have been associated with 5-FU resistance. Anthracycline doxorubicin (adriamycin) is one of known substrates for multidrug resistance (MDR1) protein (P-glycoprotein). Thus, doxorubicin resistance may occur as a consequence of P-glycoprotein overexpression or also *via* altered topoisomerase II activity. The third of FAC drugs, cyclophosphamide, belongs to the alkylating agents. At least four categories of resistance to alkylating agents are known, including increased cytosolic drug inactivation, enhanced repair of DNA damage and resistance to apoptosis [3–5].

The choice of therapy in breast cancer is based on combination of the staging system, age at diagnosis, tumor histotype and stage, histological grade, hormone receptor status and tumor-node-metastasis (TNM) staging. To offer treatment with increased efficacy and low toxicity, the development of selective therapies based on patient as well as clinical and molecular tumor characteristics is necessary. Developing such therapies should be based on the knowledge of benefits and potential acute and late toxic effects of each of the therapeutic regimens [4]. Further research is suggested regarding the effectiveness of therapy, and also the areas such as genetic markers, chemotherapy regimens, patient quality of life, and patient views on survival advantages versus treatment disadvantages [6]. Consideration of the economic assessment and the intended effects of therapy should be used for clinical decision-making [7]

The aim of our study was to analyze the possible impact of wide and comprehensive set of polymorphic variants in genes with known or potential role in the activity on FAC drugs on the prediction of long term survival of the carriers of favorable or unfavorable polymorphic variants. Analyzed SNPs (single nucleotide polymorphisms) are present in genes encoding proteins involved in FAC drugs transport (*ABCB1*, *ABCC2*, *ABCG2, SLC22A16*), metabolism (*CYP1B1*, *CYP2C19*, *GSTT1*, *GSTM1*, *GSTP1*, *TYMS*, *MTHFR*, *DPYD*), drug-induced damage repair (*ERCC1*, *ERCC2*, *XRCC1*); they are also involved in the regulation of DNA damage response and cell cycle control (*ATM*, *TP53*). The genetic variants addressed in this work had been extensively studied previously by several teams in terms of impact on protein function and treatment outcome in many conditions, including breast cancer [8–12]. These authors concluded that, apart from clinical factors, patient-related factors are of great importance for the treatment outcome. Therefore, we assumed that functional polymorphisms in genes encoding key proteins of metabolic pathways of FAC drugs, alone or in combinations, may explain some of the inter-individual variation in the treatment response in breast cancer patients. The novelty in our model is the separation of triple negative breast cancer (TNBC) patients as the subgroup with the worst prognosis. This analysis should contribute to the elucidation of the mechanisms of drug resistance in this subgroup of breast cancer patients.

## RESULTS

### Correlation between clinical and genetic factors and lack of response to FAC treatment

The data regarding treatment outcome was available for all the patients. In univariate analyses (Table 1) the strongest factor affecting treatment outcome was the presence of preexisting metastases, which significantly increased the lack of treatment response risk (OR 9.67; 95% CI 4.01-23.33; p<0.00001). The other clinical factor that tended to change the elevated risk was the nodes’ status (OR 2.08 ; 95% CI 0.91-4.71; p=0.078). Neither tumor size (T) nor triple-negativity were linked to the lack of response to FAC chemotherapy. Tumor grading, histotype, patient’ age and neo/adjuvant chemotherapy setting did not correlate with the responsiveness to treatment either (Pearson χ^2^ test p=0.364, p=0.188, p=0.471, p=0.36, respectively; data not shown).

**Table 1:** Univariate analyses of the associations between SNPs and lack of treatment response risk

Effect	Variable	Distribution analysis p	Lack of treatment response risk OR (± 95% CI)	p
Risk enhancers	***ABCC2* c.-24C>T rs717620**			
	**CC versus CT**	0.078*	0.69 (0.34-1.40)	0.307
	**CC versus TT**		**3.86** (0.82-18.15)	**0.086**
	**CC versus CT+TT**	0.628**	0.82 (0.43-1.60)	0.576
	**CC+CT versus TT**	0.078**	**4.29** (0.93-19.91)	**0.063**
	***ERCC2***p.Lys751Gln rs13181****			
	**TT versus TG**	0.031*	**1.96** (0.93-4.16)	**0.077**
	**TT versus GG**		**3.24** (1.30-8.05)	**0.011**
	**TT versus TG+GG**	0.025**	**2.23** (1.09-4.57)	**0.027**
	**TT+TG versus GG**	0.052**	**2.11** (1.00-4.44)	**0.048**
	***GSTT1***gene deletion****			
	**gene present versus gene deletion**	0.045**	**2.16** (1.08-4.32)	**0.029**
	**N (nodes)**			
	**0 versus 1-4**	0.103**	**2.08** (0.91-4.71)	**0.078**
	**M (metastases)**			
	**0 versus 1**	<0.00001**	**9.6**7 (4.01-23.33)	**<0.00001**
Risk decreaser	***ABCB1***p.Ser893Ala/Thr rs2032582****			
	**GG versus GT**	0.179*	1.40 (0.69-2.84)	0.350
	**GG versus TT**		0.44 (0.14-1.45)	0.175
	**GG versus GA**		0.72 (0.08-6.43)	0.767
	**GG versus TA**		--	--
	**GG versus other**	1.000**	1.05 (0.53-2.11)	0.872
	**GG+GT+GA versus TT+TA**	0.031**	**0.32** (0.11-0.93)	**0.036**
None	*ABCB1* p.Ile1145= rs1045642			
	CC versus CT	0.920*	1.01 (0.20-2.01)	0.992
	CC versus TT		0.87 (0.35-2.15)	0.764
	CC versus CT+TT	1.000**	0.96 (0.45-2.03)	0.959
	CC+CT versus TT	0.736**	0.87 (0.44-1.72)	0.683
	*ABCC2* p.Ile1324= rs3740066			
	GG versus GA	0.723*	0.82 (0.42-1.56)	0.537
	GG versus AA		1.14 (0.44-2.93)	0.785
	GG versus GA+AA	0.755**	0.88 (0.48-1.62)	0.674
	GG+GA versus AA	0.633**	1.27 (0.52-3.67)	0.601
	*ABCC2* p.Val417Ile rs2273697			
	GG versus GA	0.662*	1.34 (0.70-2.55)	0.370
	GG versus AA		1.01 (0.10-10.52)	0.996
	GG versus GA+AA	0.423**	0.88 (0.48-1.62)	0.674
	GG+GA versus AA	1.000**	1.27 (0.52-3.07)	0.601
	*ABCG2* p.Gln141Lys rs2231142			
	CC versus CA	1.000**	0.92 (0.42-2.01)	8.24
	CC versus AA		--	--
	*ATM* p.Asp1853Asn rs1801516			
	GG versus GA	0.765*	0.85 (0.39)1.86)	0.677
	GG versus AA		0.52 (0.06-4.20)	0.535
	GG versus GA+AA	0.716**	0.80 (0.38-1.69)	0.796
	GG+GA versus AA	1.000**	0.53 (0.07-4.30)	0.555
	*CYP1B1* p.Leu432Val rs1056836			
	CC versus CG	0.446*	0.67 (0.35-1.29)	0.232
	CC versus GG		0.67 (0.26-1.72)	0.405
	CC versus CG+GG	0.256**	0.67 (0.36-1.24)	0.205
	CC+CG versus GG	0.834**	0.85 (0.36-2.00)	0.699
	*CYP2C19* p.Pro227= rs4244285			
	GG versus GA	0.659*	0.82 (0.39-1.75)	0.608
	GG versus AA		1.78 (0.34-9.23)	0.490
	GG versus GA+AA	0.861**	0.90 (0.45-1.83)	0.776
	GG+GA versus AA	0.357**	1.86 (0.36-9.55)	0.455
	*DPYD* p.Ile543Val rs1801159			
	AA versus AG	0.807*	1.16 (0.60-2.23)	0.663
	AA versus GG		0.63 (0.08-5.17)	0.662
	AA versus AG+GG	0.745**	1.10 (0.58-2.09)	0.767
	AA+AG versus GG	1.000**	0.60 (0.07-4.87)	0.630
	*ERCC1* c.1510C>A rs3212986			
	CC versus CA	0.461*	1.43 (0.76-2.69)	0.266
	CC versus AA		1.63 (0.50-5.31)	0.419
	CC versus CA+AA	0.278**	1.46 (0.79-2.67)	0.223
	CC+CA versus AA	0.529**	1.39 (0.44-4.37)	0.570
	*ERCC1* p.Asn118= rs11615			
	TT versus TC	0.900*	0.86 (0.45-1.64)	0.650
	TT versus CC		0.95 (0.35-2.58)	0.926
	TT versus TC+CC	0.755**	0.88 (0.48-1.62)	0.680
	TT+TC versus CC	1.000**	1.03 (0.41-2.62)	0.948
	*GSTM1* gene deletion			
	gene present versus gene deletion	0.873*	0.93 (0.49-1.76)	0.823
	*GSTP1* p.Ile105Val rs1695			
	AA versus AG	0.893*	1.13 (0.60-2.14)	0.701
	AA versus GG		1.23 (0.42-3.59)	0.702
	AA versus AG+GG	0.758**	1.15 (0.62-2.11)	0.654
	AA+AG versus GG	0.788**	1.16 (0.42-3.20)	0.778
	*MTHFR* p.Ala222Val rs1801133			
	CC versus CT	0.317*	1.22 (0.65-2.29)	0.527
	CC versus TT		0.48 (0.14-1.71)	0.481
	CC versus CT+TT	1.000**	1.04 (0.57-1.92)	0.888
	CC+CT versus TT	0.233**	0.44 (0.13-1.48)	0.182
	*SLC22A16* p.Met409Thr rs12210538			
	AA versus AG	0.724*	0.79 (0.40-1.59)	0.510
	AA versus GG		1.22 (0.38-3.88)	0.736
	AA versus AG+GG	0.750**	0.86 (0.46-1.63)	0.651
	AA+AG versus GG	0.547**	1.31 (0.42-4.09)	0.641
	*SLC22A16* p. p.His49Arg rs714368			
	AA versus AG	0.648*	0.95 (0.50-1.-81)	0.872
	AA versus GG		1.82 (0.46-7.18)	0.388
	AA versus AG+GG	1.000**	1.02 (0.55-1.89)	0.947
	AA+AG versus GG	0.409**	1.86 (0.48-7.15)	0.366
	*TP53* p.Arg72Pro rs1042522			
	GG versus GC	0.881*	1.18 (0.62-2.21)	0.616
	GG versus CC		0.99 (0.36-2.72)	0.988
	GG versus GC+CC	0.648**	1.16 (0.63-2.12)	0.635
	GG+GC versus CC	1.000**	0.99 (0.36-2.72)	0.988
	*TYMS* STR 3R/2R rs34743033			
	3R3R versus 3R2R	0.426*	1.59 (0.75-3.39)	0.224
	3R3R versus 2R2R		1.64 (0.66-4.08)	0.282
	3R3R versus 3R2R+2R2R	0.238**	1.61 (0.78-3.30)	0.195
	3R3R+3R2R versus 3R2R	0.697**	1.21 (0.58-2.52)	0.616
	*XRCC1* p.Gln399Arg rs25487			
	GG versus GA	0.509*	1.39 (0.73-2.66)	0.314
	GG versus AA		0.90 (0.31-2.61)	0.848
	GG versus GA+AA	0.535**	1.28 (0.69-2.38)	0.435
	GG+GA versus AA	0.816**	0.76 (0.28-2.04)	0.579
	T (tumor)			
	0+1 versus 2-4	0.111**	2.69 (0.79-9.19)	0.111
	TNBC			
	no versus yes	1.000**	1.05 (0.41-2.69)	0.918

* Pearson χ^2^ test

** Fisher two-way exact test; OR- odds ratio; 95%CI- confidence interval; - tumor stage; N- nodes stage; M- metastases stage; TNBC- triple negative breast cancer; bolded numbers indicate results with p<0.100, significant statistical associations or trends are in bold

The polymorphisms in ERCC2 and two ABC transporter genes, as well as homozygous deletion of *GSTT1* gene changed the lack of FAC response risk. The absence of wild type allele G for trinucleotide variant p.Ser893Ala/Thr (rs2032582) in *ABCB1* gene decreased the risk to OR 0.32 (95% CI 0.11-0.93; p=0.036). For the promotor variant c.-24C>T (rs717620) in *ABCC2* gene there was a tendency towards higher risk of lack of response for rare homozygote TT when compared to wild type homozygote CC alone (OR 3.86; 95% CI 0.82-18.15; p=0.086) and to combined genotypes with wild allele (OR 4.29; 95% CI 0.93-19.91; p=0.063). Significant increase of lack of treatment response risk was observed for polymorphism p.Lys751Gln (rs13181) in *ERCC2* gene in every combination. Similarly, such significant risk increase was seen for homozygous deletion of *GSTT1* gene (OR 2.16; 95% CI 1.08-4.32; p=0.029). All above-mentioned factors that influenced the risk of lack of response to treatment with p-value under 0.100 were included into multivariate analysis. Since the A and T alleles of variant p.Ser893Ala/Thr (rs2032582) in *ABCB1* gene were the only ones that decreased the risk of unresponsiveness to FAC chemotherapy, in the next step of analyses the presence of these alleles was treated as a reference group. This enabled us to build the model of solely high-risk genotypes in multivariate analysis. After stepwise regression where the factors with the highest p-value were consecutively rejected, the final model of independent prognostic factors was established. In this model the factors responsible for high lack of response risk were: the presence of preexisting metastases (OR 17.34; 95% CI 6.02-49.93; p<0.00001), the rare homozygote TT of *ABCC2* promoter polymorphism c.-24C>T (rs717620) (OR 8.84; 95% CI 1.11-70.48; p=0.039) and the genotypes with the rare G allele of *ERCC2* p.Lys751Gln (rs13181) variant (OR 4.51; 95% CI 1.72-11.82; p=0.002). The genotypes containing wild G allele of p.Ser893Ala/Thr (rs2032582) polymorphism in *ABCB1* gene were responsible in multivariate analysis for increased risk at borderline statistical significance (OR 3.19; 95% CI 0.98-10.39; p=0.053) (Table 2).

**Table 2:** Multivariate analysis of the associations between SNPs and lack of treatment response risk

Variable	Lack of treatment response		Lack of treatment response risk OR (± 95% CI)	p
	no n (%)	yes n (%)		
*ABCB1* p.Ser893Ala/Thr rs2032582				
TT+TA	58 (21.3)	4 (8.0)	1 (ref)	
GG+GT+GA	214 (78.7)	46 (92.0)	3.19 (0.98-10.39)	0.053
*ERCC2* p.Lys751Gln rs13181				
TT	104 (38.7)	11 (22.0)	1 (ref)	
TG+GG	165 (61.3)	39 (78.0)	**4.51** (1.72-11.82)	**0.002**
*ABCC2* c.-24C>T rs717620				
CC+CT	269 (98.5)	47 (94.0)	1 (ref)	
TT	4 (1.5)	3 (6.0)	**8.84** (1.11-70.48)	**0.039**
M (metastases)				
0	228 (95.4)	30 (68.2)	1 (ref)	
1	11 (4.6)	14 (31.8)	**17.34** (6.02-49.93)	**<0.00001**

OR- odds ratio; 95%CI- confidence interval; associations in bold indicate results with p<0.05

### Cumulative analysis of genetic factors and lack of response to FAC treatment

In the next step cumulative analysis was performed to study the effect of simultaneous presence of one, two, or all three unfavorable genotypes of genetic polymorphisms established in multivariate analysis as independent prognostic factors. It should be noted that in the studied group there were no cases of lack of treatment response in the group of patients that had none of the unfavorable genotypes (i.e. the 0's group). Therefore, the lack of treatment response risk analyses were conducted with a combined group of 0's and 1's as the reference. The results show that the growing number of high-risk genotypes is reflected in the gradual risk increase of the non-responsiveness to treatment - from OR 2.68 (95% CI 1.37-5.23; p=0.004) for the presence of two genotypes to OR 9.93 (95% CI 1.28-77.25; p=0.027) for the carriers of all three negative genotypes. Also, the combined analysis done for the group carrying more than one high-risk genotype revealed nearly 3-fold higher risk of lack of response (OR 2.79; 95% CI 1.44-5.43; p=0.002) when compared to the reference group (Table 3).

**Table 3:** The association between accumulation of unfavorable genotypes and lack of treatment response risk

		ALL PATIENTS					TNBC's SUBGROUP				
		Lack of treatment response			Lack of treatment response risk OR (± 95% CI)	p	Lack of treatment response			Lack of treatment response risk OR (± 95% CI)	p
Unfavorable genotypes	Number of unfavorable genotypes	no n (%)	yes n (%)	p			no n (%)	yes n (%)	p		
*ABCB1* p.Ser893Ala/Thr rs2032582 GG/GT/GA	0	20 (7.5)	0 (0.0)	0.003*	1 (ref)		1 (3.3)	0 (0.0)	0.067*	1 (ref)	
	1	119 (44.6)	14 (28.0)				16 (53.3)	1 (16.7)			
*ERCC2* p.Lys751Gln rs13181 TG/GG	2	126 (47.1)	34 (68.0)		**2.68** (1.37-5.23)	**0.004**	12 (40.0)	3 (50.0)		4.25 (0.36-50.62)	0.234
	3	2 (0.8)	2 (4.0)		**9.93** (1.28-77.25)	**0.027**	1 (3.3)	2 (33.3)		**34.0** (1.20-967.50)	**0.028**
*ABCC2* c.-24C>T rs717620 TT	0+1	139 (52.1)	14 (28.0)	0.002**	1 (ref)		17 (56.7)	1 (16.7)	0.177**	1 (ref)	
	2+3	128 (47.9)	36 (72.0)		**2.79** (1.44-5.43)	**0.002**	13 (43.3)	5 (83.3)		6.54 (0.62-68.5)	0.104

* Pearson χ^2^ test

** Fisher two-way exact test; OR- odds ratio; 95% CI- confidence interval; associations in bold indicate results with p<0.05; TNBC- triple negative breast cancer

To clarify whether the group with the worst prognosis (i.e. the 3′s) was or was not composed of patients with clinical factors responsible for bad treatment outcome, correlations were performed between the groups 0-3 and clinical factors established by cumulative analysis, in order to obtain more exact characterization of these groups (Table 4). The results indicate that there was a strong correlation with triple negative tumors (p=0.0009), which were more common in patients carrying three unfavorable genotypes - 75% versus 5.3%, 13.2% and 10.1% for the 0-2 groups, respectively. The other strongly significant correlation was seen for tumor histotype (p=0.007). Groups with one to two unfavorable genotypes were composed mostly of the invasive ductal carcinoma subtype (90.0%, 69.7% and 75.0%), while in the group with three variants there was no predominant subtype. The borderline significance was detected for estrogen receptor status and tumor grade (p=0.063 and 0.056). It should be noted that there was no statistically significant correlation between PR and HER2 status when analyzed separately, nor between TNM staging and age at the time of diagnosis either. Because the strongest correlation for the cumulative groups was detected for triple negative cancers (p=0.0009) we performed cumulative analysis again for the TNBCs alone (Table 3). Once again the group of 0's showed no cases of lack of treatment response and the combined group of 0's and 1's was used as a reference. These results indicated that there was a subgroup of triple negative breast cancers, carriers of all three high-risk genotypes, that harbored an extremely high risk of FAC treatment non-responsiveness (OR 34.0; 95% CI 1.20-967.50; p=0.028).

**Table 4:** Clinical characteristics of cumulative groups

Variable	Cumulative groups - number of unfavorable genotypes n(%)				Distribution analysis p
	0	1	2	3	
**ER**					
negative	8 (42.1)	44 (34.6)	57 (38.5)	4 (100.0)	0.063**
positive	11 (57.9)	83 (65.4)	91 (61.5)	0 (0.0)	
**PR**					
negative	7 (36.8)	55 (43.3)	65 (43.9)	4 (100.0)	0.138**
positive	12 (63.2)	72 (56.7)	83 (56.1)	0 (0.0)	
**HER2**					
negative	5 (29.4)	37 (33.3)	55 (41.7)	3 (75.0)	0.200**
positive	12 (70.6)	74 (66.7)	77 (58.3)	1 (25.0)	
**TNBC**					
no	18 (94.7)	112 (86.8)	133 (89.9)	1 (25.0)	0.0009**
yes	1 (5.3)	17 (13.2)	15 (10.1)	3 (75.0)	
**G**					
1	2 (11.8)	19 (20.9)	17 (15.7)	---	
2	5 (29.4)	27 (29.7)	38 (35.2)	---	
3	8 (47.0)	33 (36.2)	40 (37.0)	1 (50.0)	0.056*
Bloom I	---	1 (1.1)	2 (1.9)	1 (50.0)	
Bloom II	1 (5.9)	6 (6.6)	5 (4.6)	---	
Bloom III	1 (5.9)	5 (5.5)	6 (5.6)	---	
**T**					
0+1	1 (5.3)	17 (15.3)	24 (16.9)	0 (0.0)	0.549**
2-4	18 (94.7)	94 (84.7)	118 (83.1)	2 (100.0)	
**N**					
0	4 (21.0)	33 (29.7)	46 (32.4)	1 (33.3)	0.782**
1-4	15 (79.0)	78 (70.3)	96 (67.6)	2 (66.7)	
**M**					
0	16 (84.2)	100 (89.3)	133 (93.0)	2 (100.0)	0.502**
1	3 (15.8)	12 (10.7)	10 (7.0)	---	
**Histopahology**					
invasive ductal carcinoma	18 (90.0)	92 (69.7)	120 (75.0)	1 (25.0)	
invasive lobular carcinoma	1 (5.0)	13 (9.9)	9 (5.6)	0 (0.0)	0.007*
carcinoma mixed type	0 (0.0)	4 (3.0)	1 (0.6)	1 (25.0)	
other	0 (0.0)	12 (9.1)	7 (4.4)	1 (25.0)	
unspecified	1 (5.0)	11 (8.3)	23 (14.4)	1 (25.0)	
**Age at diagnosis** (years)					
≤39	---	10 (7.5)	15 (9.4)	---	0.320*
40-60	17 (85.0)	86 (64.7)	111 (69.4)	4 (100.0)	
≥61	3 (15.0)	37 (27.8)	34 (21.2)	---	

* Pearson χ^2^ test

** Fisher two-way exact test; associations in bold indicate results with p<0.05; ER- estrogen receptor; PR- progesterone receptor; HER2- human epidermal growth factor receptor-2; TNBC- triple negative breast cancer; T- tumor stage; N- nodes stage; M- metastases stage

### Potential application of cumulative model – survival analysis

The definition of treatment failure in our study was based on the events that occurred within 10 months from the beginning of FAC chemotherapy. To check possible application of cumulative model for predicting long-term survival of patients, the TFFS (treatment failure-free survival) analysis was performed. Similarly as in cumulative analyses, the group lacking unfavorable genotypes had the best outcome – there were no events such as disease progression, local recurrence, metachronous breast cancer, or death in follow-up. For this reason (censored observations) in Cox proportional hazard calculations of treatment failure risk the reference group was the one carrying one high-risk genotype.

**Figure 1:**
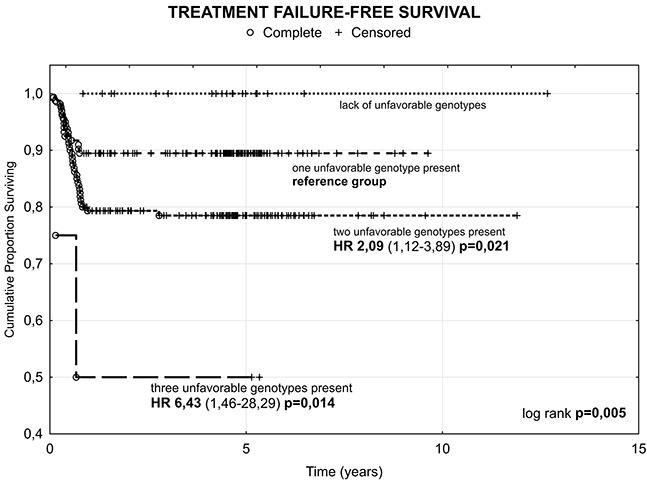
Kaplan-Meier analysis of Treatment Failure-Free Survival (TFFS) of breast cancer patients according to number of unfavorable genotypes HR- hazard ratio.

The results showed the shortening of TFFS with the increasing number of high-risk genotypes, with median TFFS of 54.4 months for carriers of one variant, to 51.5 and 34.9 months for the carriers of two and three genotypes, respectively. Cox proportional hazard analysis showed also consecutive increase of the risk of treatment failure from HR 2.09 (95% CI 1.12-3.89; p=0.021) for the presence of two unfavorable genotypes to HR 6.43 (95% CI 1.46-28.29; p=0.014) for all three genotypes (Figure 1).

## DISCUSSION

In this study, we genotyped 22 variants in 15 genes belonging to the main pathways and cellular mechanisms engaged in transport and activity of three FAC drugs in order to select genetic changes that are linked to unfavorable reaction to treatment. Our results show that the risk of lack of response is modified by variability in genes engaged in drugs’ transport (*ABCB1*, *ABCC2*) and in DNA repair machinery (*ERCC2*). These findings are consistent with the current knowledge. Several mechanisms are thought to be responsible for resistance to systemic breast cancer therapy, e.g. decreased drug accumulation and drug activation, increased inactivation of drug or its intermediates. Treatment effectiveness is also decreased by the enhanced DNA repair, impaired recognition of DNA adducts and defective cell cycle checkpoints resulting in the increased tolerance to drug-induced damage. Equally important are host-drug interactions, which include drug activation/inactivation by normal tissues, as well as toxic reactions with normal tissues [4]. The activity of transport proteins is emphasized as one of the crucial factors of drugs’ bioavailability and disposition in cancer patients because of the narrow therapeutic index of many cytotoxic agents [13]. In our study group the modifications in two ABC transporters, ABCB1 and ABCC2, were linked to unfavorable outcome. This result provides further emphasis on the role of cellular efflux systems in the treatment response. Among the components of this system the P-glycoprotein encoding gene (ABCB1) is regarded as the main contributor of multidrug resistance due to a vast array of substrates, with many chemotherapeutics included like anthracyclines, taxanes and Vinca alkaloids. P-glycoprotein is highly expressed in apical surface of epithelial cells in the colon and small intestine, bile ductules, kidney proximal tubules and at the blood-tissue barriers. Its localization confirms the crucial role in the systemic protection against endogenous compounds and xenobiotics [14]. Similarly, the ABCC2 transporter is highly expressed on the apical sides of polarized cells in the liver, kidneys and small intestine, but not at the blood-tissue barriers. ABCC2 is involved in excretion of many compounds like glutathione, its conjugates, folates and organic anions, mainly with bile. Many chemotherapeutics are ABCC2 substrates, including doxorubicin, epirubicin, methotrexate, cisplatin, etoposide, irinotecan and vincristine [15–17]. In our study group, variation in the *ERCC1* (also xeroderma pigmentosum group D - *XPD*) gene was the third independent factor of treatment responsiveness. *ERCC2* encodes a highly conserved ATP-dependent helicase which is a key rate-limiting protein of the NER (nucleotide excision repair) machinery. It also takes part in basal transcription being a part of transcription factor complex. The crucial function of NER system is the removal of DNA adducts and crosslinks. These lesions may be caused by many environmental factors (e.g. UV, tobacco smoke), as well as by the exposure to many cytotoxic drugs, like cisplatin or cyclophosphamide. Because of the important role of ERCC2 it is believed that even mild modifications of its sequence or structure may be significant for DNA repair efficiency, cancer risk and clinical effects of cytotoxic treatment [18–20].

Our results of cumulative analyses further emphasize the importance of changes that occur simultaneously in cellular transport (p.Ser893Ala/Thr in *ABCB1*, c.-24C>T in *ABCC2*) and DNA repair systems (p.Lys751Gln in *ERCC2*) for the outcome of treatment in breast cancer patients. It is quite plausible that only the accumulation of many, individually weak, genetic changes is strong enough to influence the reaction at the whole body level. There have been a few reports describing the concurrent impact of several SNPs on the treatment outcome in breast cancer patients. The group of Bewick [19] constructed the model consisting of three polymorphisms in genes of the NER repair system - *ERCC1* C8092A (rs3212986) and *ERCC2* p.Lys751Gln (rs13181) and p.Asp312Asn (rs1799793). They reported significant decrease of median BCSS and PFS with growing number of adverse genotypes. For the PFS analysis a similar trend was observed. The worst prognosis, similarly to our study, concerned the group carrying all three unfavorable genotypes as compared to the group lacking these alleles. This effect was seen in the patients’ group treated with a combination of cyclophosphamide, mitoxantrone and vinblastine, as well as in the group receiving cyclophosphamide, mitoxantrone and carboplatin. The importance of the impact of modifications in different pathways on treatment outcome in breast cancer was reported by Tengström et al. [21]. Their cumulative model was constructed from polymorphism p.Lys751Gln (rs13181) in *ERCC2* and p.Val16Ala in antioxidant enzyme gene *SOD2*. The patients with one or two low-risk genotypes had improved recurrence-free survival (RFS), breast cancer specific survival (BCSS) and overall survival (OS). It should be noted that in both cited studies, similar to ours, the wild homozygote TT of the p.Lys751Gln polymorphism was at lower risk of treatment failure when compared to other genotypes. Our cumulative model emphasized that, for the desired treatment outcome, the activity of export systems through the ABC transporters and capability to repair drug-caused DNA damage were essential. Furthermore, the correlation analyses revealed that in the group with the worst prognosis the triple negative breast cancers (TNBC) are more common than in other groups. TNBCs are negative for estrogens receptor (ER), progesterone receptor (PR) and human epidermal growth factor receptor 2 (HER2), and are well known for aggressive behavior and distinct metastatic patterns. These tumors are characterized by onset at a younger age, high mean tumor size, higher-grade tumors and sometimes a higher rate of node positivity. Although triple negative breast cancers are associated with a generally poor breast cancer specific outcome, most are not resistant to standard chemotherapy regimens, but due to their unique molecular profile and heterogeneity, the development of targeted therapies is needed [22–24]. Because of the overrepresentation of TNBCs in the group with worst prognosis in this study, we wanted to see if our cumulative model was applicable also to the separation of heterogeneous TNBCs into subgroups with different prognoses. The unfavorable cumulative effect was statistically significant for carriers of all three high-risk variants, which might indicate existence of a specific molecular subgroup of TNBCs. Similar to the analysis made for all patients, the group lacking the high-risk genotypes had no cases of lack of treatment response. A recent report by Le Du et al. [23] showed a classification based on molecular subtypes of TNBC. The authors distinguished five possible subgroups, each of them defined by a dominant biological function or pathway. They emphasized the need of developing a set of biomarkers relevant for TNBCs included in the treatment options and serving as the tool to overcome resistance to proposed treatment. Our study indicated the existence of chemo-resistant subpopulation of TNBCs characterized by altered efflux and DNA repair systems. Le Du et al. concluded that the preselection of chemo-resistant population of TNBC is necessary, as such group can benefit from additional treatment options. It should be noted that, at present, there are no specific treatment guidelines for TNBC subgroup and that these cancers are managed with standard treatment [25].

The potential usefulness of our model seems to be further confirmed by the analysis of TFFS. One should note that the groups of responders and non-responders to FAC regime and, consequently, all predictive factors were distinguished by the unfavorable events that occurred within 10 months from the beginning of treatment. In these settings the developed cumulative model still enabled us to significantly predict the long-term survival of the patients, and still the prognosis for the group with three high-risk genotypes was the worst. At the same time, the group lacking these genotypes registered no case of disease progression, local recurrence, metachronous breast cancer, or death in the follow-up.

As we stated above, worse response to treatment observed in our cumulative analyses for the carriers of two and three unfavorable alleles could be the result of more intense activity of transporters and, consequently, higher drugs’ clearance combined with improved DNA repair machinery. Such situation occurs with the presence of alanine in position 893 (G allele) of p.Ser893Ala/Thr (rs2032582) *ABCB1* variant, T allele of c.-24C>T (rs717620) *ABCC2* promoter variant and with the presence of glutamine in position 751 (G allele) of p.Lys751Gln (rs13181) *ERCC2* polymorphism. However, the exact functions of these polymorphic variants remain unclear, for the results and conclusions coming from different papers are often contradictory. The trinucleotide change p.Ser893Ala/Thr (rs2032582) is undoubtedly one of the most studied variants of *ABCB1* gene; similarly, much attention has been lately given to the *ABCC2* promoter polymorphism c.-24C>T. The lack of conclusion regarding their functions appears to result from many factors, including kind of substrate, characteristics of the studied group and ethnicity. Our hypothesis that G allele of *ABCB1* rs2032582 variant is linked to improved drug efflux has found support in the study of Kim et al. [26], who evaluated activity of this transporter's different alleles in oocytes of *X. laevis*. The authors showed that allele A (threonine) is responsible for much slower transport of digoxin and daunorubicin when compared to alleles G (alanine) and T (serine). Consequently, the genotypes with the G allele are characterized by more efficient removal of ABCB1 substrates from the cell. Such transporter modification could therefore greatly decrease drugs’ concentration in the tissues and limit their therapeutic potential. Simon et al. [27] studied the impact of *ABCC2* polymorphisms in the patients with lymphoid malignancies receiving high-dose methotrexate. In this study, the presence of T allele of c.-24C>T polymorphism was the only predictor of methotrexate clearance and volume of distribution; consequently, the T allele carriers were more efficient in methotrexate removal from the body. These results remain in accordance with our observations that high risk of lack of response to treatment detected for the carriers of rare TT homozygotes could be explained by more efficient excretion of FAC drugs being the ABCC2 substrates. The *ERCC2* p.Lys751Gln polymorphism had been extensively studied, among a few others, as the possible risk factor of developing malignancy, as well as the modifier of treatment efficiency. It is believed that glutamine replacement by lysine completely changes charge configuration in the protein molecule, and this could be significant given the location of the variant at the domain important for interaction with helicase activator [20, 28] As stated before, our results lead to hypothesis that the repair capacity of ERCC2 protein with Gln751 is improved when compared to that of wild-type T allele and, therefore, the efficiency of cytotoxic agents becomes limited. This assumption finds confirmation in the work of Lunn et al. [29], who measured the frequency of X-ray induced chromatid aberration in the presence of different p.Lys751Gln alleles. Their results show that aberrations’ frequency for Lys/Lys is higher than that for Gln/Gln and, consequently, the risk of sub-optimal DNA damage repair is also elevated. Similar observations were made by Bewick et al. [19] who concentrated on the relations between SNPs in NER components and outcome of patients with metastatic breast cancer. They showed that the breast cancer specific survival was significantly better for the wild-type T allele carriers, and the rare GG homozygote was considered as adverse genotype. Thus, the results of Bewick et al. [19] stay in accordance with our observations that the combined group of TG and GG carriers was at high risk of lack of response to treatment. It should be also noted that in our analysis the p.Lys751Gln variant was the prognostic factor independent from preexisting metastases (M+). Improved survival for the wild TT homozygotes in breast cancer patients was reported also by Chew et al. [30] for the gemcitabine/cisplatin regime and by Tengstrom et al. [21] for the patients treated with tamoxifen.

In conclusion, we found significant association between concurrent polymorphic variants of genes responsible for drug transport and DNA repair and the responsiveness of the breast cancer patients to FAC chemotherapy. Our results also demonstrate the existence of strong gene-dosage effect, where the risk of lack of response to treatment is gradually rising with the number of unfavorable alleles. The findings also demonstrate that the results of cancer treatment are a combined effect of numerous factors, including clinical characteristics of the disease and the common interindividual genetic variation. It seems that multifactorial polymorphic models could be useful tools in personalizing cancer therapies. The presented models could be useful in separating heterogenous groups of patients sharing general diagnosis into more uniform subpopulations that could benefit from different or just slightly modified treatment strategies.

## MATERIALS AND METHODS

### Patients and samples

A total of 324 women from the Silesian voivodeship (southern Poland), diagnosed with breast cancer were recruited to this study. Cases with DCIS (ductal carcinoma *in situ*) or LCIS (lobular carcinoma *in situ*), as well as Paget tumors were excluded. All study subjects were treated with FAC first-line chemotherapy regime which combines doxorubicin (50mg/m^2^), 5-fluorouracil (500 mg/m^2^) and cyclophosphamide (500 mg/m^2^). At the Cancer Center in Gliwice only the patients who were diagnosed with cardiovascular disease or with low left ventricular ejection fraction did not qualify for FAC treatment. The drugs were administered intravenously on the first day of 21-day cycle; there were six planned cycles for each patient, in adjuvant or neoadjuvant setting. The status of the Silesian most common germline mutations in *BRCA1* and *BRCA2* genes (c.68_69delAG, c.181T>G, c.4034delA, c.5266dupC in *BRCA1* and c.5946delT, c.9403delC in *BRCA2* gene) was analyzed for all the patients in this study. The chosen group was composed only of non-carriers. All patients filled the informed consent form and agreed to have their samples used for research purposes. This study was approved by the local Bioethical Committee.

Full characteristics of the group under study is given in Table 5. The observation ended the 1^st^ of March 2015.

**Table 5:** Characteristics of breast cancer patients under study group

	Charateristics	n (%)
**GENERAL**	**Age at diagnosis** (years)	
	• ≤39	26 (8.0)
	• 40-50	222 (68.5)
	• ≥61	76 (23.5)
	Mean age at diagnosis in years (min-max)	54.7 (22.4-79.0)
	**Year of diagnosis**	
	• 1997-2004	15 (4.6)
	• 2005-2009	289 (89.2)
	• 2010-2012	20 (6.2)
	**Histopathology**	
	• invasive ductal carcinoma	230 (71.1)
	• invasive lobular carcinoma	22 (6.8)
	• carcinoma mixed type	6 (1.8)
	• other	29 (8.9)
	• unspecified	37 (11.4)
	**Tumor grade**	
	• G1	39 (12.0)
	• G2	71 (21.9)
	• G3	83 (25.8)
	• Bloom I	5 (1.5)
	• Bloom II	12 (3.7)
	• Bloom III	12 (3.7)
	• unspecified	102 (31.4)
**RECEPTORS**	**Estrogen receptor status**	
	• negative	115 (35.5)
	• positive	190 (58.6)
	• unspecified	19 (5.9)
	**Progesterone receptor status**	
	• negative	133 (41.0)
	• positive	172 (53.1)
	• unspecified	19 (5.9)
	**HER2 status**	
	• negative	103 (31.8)
	• positive	167 (61.5)
	• unspecified	54 (16.7)
	**triple-negative breast cancer (TNBC)**	37 (11.4)
**TNM staging**	**Tumor (T)**	
	• 0	2 (0.6)
	• 1	43 (13.3)
	• 2	97 (29.9)
	• 3	52 (16.0)
	• 4	82 (25.3)
	• unspecified	50 (15.5)
	**Nodes (N)**	
	• 0	85 (26.2)
	• 1	108 (33.3)
	• 2	67 (20.7)
	• 3	19 (5.8)
	• 4	1 (0.3)
	• inspecified	44 (13.6)
	**Metastases (M)**	
	• 0	258 (79.6)
	• 1	25 (7.7)
	• unspecified	42 (12.7)
	**Metastases locations**	
	• liver	8 (32.0)
	• lungs	3 (12.0)
	• bones and lungs	3 (12.0)
	• bones	2 (8.0)
	• other	9 (36.0)
**THERAPY**	**Surgery**	
	• amputation	187 (57.7)
	• conserving surgery. including:	87 (26.8)
	• with radicalization	14 (4.3)
	• without radicalization	73 (22.5)
	• none	50 (15.5)
	**Hormonotherapy**	
	• yes	204 (63.0)
	• no	120 (37.0)
	**Immunotherapy (Herceptine)**	
	• yes	36 (11.1)
	• no	288 (88.9)
	**Chemotherapy FAC**	
	• adjuvant	136 (42.0)
	• neoadjuvant	188 (58.0)
	mean numer of cycles (range)	6.1 (3-9)
	**Radiotherapy**	
	• yes	265 (81.8)
	• no	59 (18.2)
	• brachytherapy	7 (2.2)
	mean radiation dose in Gy (range)	50.2 (20-70)
	mean radiation dose in brachytherapy (range)	14 (10-30)
**FOLLOW-UP**	**Deaths**	
	• yes	85 (26.2)
	• no	239 (73.8)
	median OS in months (min-max)	57.6 (4.3-156.2)
	**Progression**	
	• yes	99 (30.6)
	• no	225 (69.4)
	median PFS in months (min-max)	54.1 (0.9-152.1)
	**Progression- locations of metastases**	
	• bones	28 (28.3)
	• multiorgan spread	27 (27.3)
	• lungs	8 (8.1)
	• liver	8 (8.1)
	• lymph nodes	8 (8.1)
	• tumor growth	8 (8.1)
	• central nervous system	7 (7.1)
	• skin	4 (4.0)
	• eye socket	1 (1.0)
	**Recurrence**	
	• yes	12 (3.7)
	• no	312 (96.3)
	median RFS in months (min-max)	55.0 (0.5-152.1)
	**Metachronous primary breast cancer**	
	• yes	8 (2.5)
	• no	316 (97.5)
	median survival to next breast cancer diagnosis in months (min-max)	55.0 (0.5-152.1)

HER2- human epidermal growth factor receptor-2; PFS- progression-free survival; RFS- recurrence-free survival

### Genotyping

Genomic DNA was isolated from the peripheral blood leukocytes using the phenol-chloroform method or commercial DNA isolation kits. Genotyping was performed using RFLP-PCR, multiplex-PCR or allele-specific amplification PCR (ASA-PCR) methods. PCR reagents purchased from Applied Biosystems (AmpliTaq Gold Polymerase) and EURx (Perpetual Taq Polymerase) were used. Genotyping of polymorphic variants in *ABCB1* (rs1045642), *ABCC2* (rs2273697, rs717620, rs3740066), *GSTP1* (rs1695), *CYP1B1* (rs1056836), *CYP2C19* (rs4244285), *TYMS* (rs34743033), *ERCC1* (rs11615, rs3212986), *ERCC2* (rs13181), *XRCC1* (rs25487), *TP53* (rs1042522), as well as detection of GSTT1/M1 deletions were performed as described previously [31–42]. The genotyping methods for polymorphisms in *ABCB1* (rs2032582), *ABCG2* (rs2231142), *MTHFR* (rs1801133), *SLC22A16* (rs714368, rs12210538), *DPYD* (rs1801159) and *ATM* (rs1801516) were developed for this study. Primers were designed with Primer3 web application (http://primer3plus.com/) or extracted from the literature. RFLP methods including restriction sites implementation were designed using the WatCut online tool (http://watcut.uwaterloo.ca/). Primer sequences and expected amplification products were verified using NCBI BLAST tools (http://blast.ncbi.nlm.nih.gov/Blast.cgi) and *in silico* PCR (http://genome.ucsc.edu/cgi-bin/hgPcr). PCR products were digested with appropriate restriction enzymes according to manufacturers’ recommendations. PCR or digestion products were separated on agarose gels stained with ethidium bromide. For the quality control randomly selected samples of each genotype were checked by direct sequencing with full result concordance. Genotyping methods are summarized in Table 6.

**Table 6:** Genotyping methods

Category	Gene	Ref SNP ID	Alleles wt/v	Mutation	Method	Enzyme	Primer sequences 5′→3′	Method source
transporters	*ABCB1*	rs1045642 rs2032582	C/T G/T/A	p.Ile1145= p.Ser893Ala/Thr	RFLP RFLP RFLP	MboI RsaI (vA) BbvI (vT)	F: ttgatggcaaagaaataaagc; R: cttacattaggcagtgactcg Fcommon: agcaaatcttgggacaggaa; R_A_: agtccaagaactggctttgc Fcommon: agcaaatcttgggacaggaa; R_T_: tat ttagtttgactcaccttc**G**ca	[32] own
	*ABCC2*	rs2273697 rs717620 rs3740066	G/A C/T A/G	p.Val417Ile c.-24C>T; 5′-UTR p.Ile1324=	RFLP RFLP RFLP	NcoI BbsI SfaNI	F: ggcaaagaagtgtgtggat; R: acatcaggttcactgtttctcc**C**a F: taaatggttgggatgaaagg; R: gctttagaccaattgcacatc F: tggctgctatccttccctct; R: ctcagagggatcacttgtg**G**ca	[39] [39] [39]
	*ABCG2*	rs2231142	C/A	p.Gln114Lys	ASA PCR	–	F: tagcaggctttgcagaca t; R: caagccacttttctcattgtt R_C_: gaagagctgctgagaactgtaag; R_A_: cgaagagctgctgagaactt	own
	*SLC22A16*	rs714368 rs12210538	A/G A/G	p.His49Arg p.Met409Thr	RFLP RFLP	FokI StyI	F: tggagacccttcaaatttgct; R: gggcctgcagaca**G**ga F:ccaggttaggcttttctttt; R:ttgctcaatgacaggtgtag	own own
drugs metabolizers	*DPYD*	rs1801159	A/G	p.Ile543Val	RFLP	PsiI	F:ttttgcagtcacaatatgga; R:tcaaaagctcttcgaatcat	own
	*MTHFR*	rs1801133	C/T	p.Ala222Val	RFLP	TaqI	F: tgaaggagaaggtgtctgcggga; R: aggacggtgcggtgagagtg	own
	*GSTT1*	--	+/−	gene deletion	multiplex PCR	–	T1-F: tctccttactggtcctcacatctc; T1-R: tcaccggatcatggccagca M1-F: gaactccctgaaaagctaaagc; M1-R: gttgggctcaaatatacggtg β-globin-F: gaagagccaaggacaggtac; β-globin-R: caacttcatccacgttcacc	[42]
	*GSTM1*	--	+/−	gene deletion				
	*GSTP1*	rs1695	A/G	p.Ile105Val	RFLP	Alw26I	F: accccagggctctatgggaa; R: tgagggcacaagaagcccct	[33]
	*CYP1B1*	rs1056836	C/G	p.Leu432Val	RFLP	AcuI	F: gcctgtcactattcctcatgcc; R: gtgagccaggatggagatgaag	[35]
	*CYP2C19*	rs4244285	G/A	p.Pro227=	RFLP	MspI	F: aattacaaccagagcttggc; R: tatcactttccataaaagcaag	[37]
5-FU target	*TYMS*	rs34743033	2R/3R	28bp tandem repeat	PCR	–	F: gtggctcctgcgtttccccc; R: gctccgagccggccacaggca	[31]
DNA repair	*ATM*	rs1801516	G/A	p.Asp1853Asn	RFLP	Sau3AI	F: taatatgtcaacggggcatg; R: atttctccatgattcatttg**G**at	own
	*ERCC1*	rs11615 rs3212986	T/C C/A	p.Asn118= c.1510C>A	RFLP RFLP	BsrDI MboII	F: aggaccacaggacacgcaga; R: catagaacagtccagaacac F: cagagacagtgccccaagag; R: gggcaccttcagctttcttt	[41] [34]
	*ERCC2*	rs13181	T/G	p.Lys751Gln	RFLP	PstI	F: ccccctctccctttcctctgttc; R: ggacctgagcccccactaacg	[36]
	*XRCC1*	rs25487	G/A	p.Arg399Glu	RFLP	MspI	F: ttgtgctttctctgtgtcca; R: tcctccagccttttctgata	[38]
	*TP53*	rs1042522	G/C	p.Arg72Pro	RFLP	Bsh1236I	F: tcccccttgccgtcccaa; R: cgtgcaagtcacagactt	[40]

bolded bases in capital letters indicate introduction of restriction site; wt- wild type; v- variant

### Study endpoints

The primary endpoint of this study was to identify the genetic and clinical determinants of non-responsiveness to FAC chemotherapy in breast cancer patients. Lack of response was defined by the presence of unfavorable events such as disease progression, local recurrence, metachronous breast cancer or death within 10 months from the beginning of chemotherapy. The selected time period enabled us to analyze the above-mentioned events that occurred during chemotherapy as well as during the 6-month period that followed chemotherapy completion. Group of non-responders consisted of 50 (15.4%) women. Several factors were considered as the determinants of unfavorable outcome, either related to tumor (TNM staging, triple negative tumors, tumor grade, histotype) or patients’ status (age at the time of diagnosis).

Overall survival (OS) was calculated as time (months) from diagnosis (established as the PCI date) to death from any cause, or to date of last contact with the patient. Median overall survival was 57.6 months; during the observation 85 patients (26.2%) from the studied group died. Progression-free survival (PFS) and recurrence-free survival (RFS) were established as time (months) from the date of first course of chemotherapy to date of progression or recurrence (confirmed by the MRI, CT or ultrasound), respectively, or to date of the last contact with the patient. Progression was confirmed in 99 (30.6%) patients in median time of 54.1 months, while the local recurrence was seen in 12 (3.7%) cases and median RFS was 55.0 months. The time to diagnosis of metachronous breast cancer was calculated as the time from the date of first course of chemotherapy to the date of PCI of the second tumor or to the last contact with the patient. Time to the development of the following breast cancer was 55.0 months; such diagnosis applied to 8 (2.5%) women (Table 5).

Treatment failure-free survival (TFFS) was calculated as time (months) from the beginning of chemotherapy (date of first course) to disease progression, recurrence, metachronous breast cancer or death, or to the date of the last contact with the patient. Median TFFS was 53.5 months.

### Statistics

The difference between observed and expected genotype frequencies in control and patient groups were tested for Hardy-Weinberg Equilibrium (HWE) using the χ^2^ test. For all studied genetic variants the genotype frequencies were in Hardy-Weinberg equilibrium and consistent with published frequencies for Caucasian population. The only deviation from HWE was the variant p.Asp1853Asn (rs1801516) in ATM gene (p=0.041; data not shown). Correlations between clinical factors, polymorphic variants and the responsiveness to the treatment were calculated with Pearson χ^2^ and Fischer two-way exact tests. A dominant, recessive and co-dominant genetic models were used in all analyses for all the genetic variants. For the p.Gln141Lys (rs2231142) polymorphism in *ABCG2* gene, we did not find any cases of rare AA homozygote in our group. Therefore, all calculations done for this variant compared wild type homozygote CC with heterozygote CA. p<0.05 was considered as statistically significant, while p<0.100 was treated as indicator for trend in given analysis.

Factors that correlated with treatment response in univariate analyses with p-value below the 0.100 were included into multivariate analyses. This cut-point was used to include into the model polymorphic variants with possible but weak impact on treatment result. The final model of independent prognostic factors (p<0.05) of FAC chemotherapy responsiveness was established after stepwise regression. Cumulative analyses were performed for the risk of lack of response to treatment connected with the concurrent presence of one and more independent prognostic factors. Risk analyses were performed using logistic regression model where odds ratios (ORs), 95% confidence intervals (95% CIs) and p values were calculated.

Survival curves were derived by Kaplan-Meier method, p values were computed by log-rank test. The relative risk of death and progression was estimated as hazard ratios (HRs), 95% confidence intervals (95% CIs) and p value by Cox proportional hazard regression model. All statistical calculations were performed using Statistica v.10.0 software (StatSoft).

## References

[1] Luqmani Y. A. (2005). Mechanisms of drug resistance in cancer chemotherapy. Med. Princ. Pract..

[2] Mitrus I, Szala S (2009). Chemotherapy – main causes of failure. Nowotwory Journal of Oncology.

[3] Klein I, Sarkadi B, Váradi A (1999). An inventory of the human ABC protein. Biochim Biophs Acta.

[4] Gonzalez-Angulo AM, Morales-Vasquez F, Hortobagyi GN (2007). Overview of resistance to systemic therapy in patients with breast cancer. Adv Exp Med Biol.

[5] Wiechec E, Hansen LL (2009). The effect of genetic variability on drug response in conventional breast cancer treatment. Eur J Pharmacol.

[6] Garside R, Pitt M, Anderson R, Rogers G, Dyer M, Mealing S, Somerville M, Price A, Stein K (2007). The effectiveness and cost-effectiveness of carmustine implants and temozolomide for the treatment of newly diagnosed high-grade glioma: a systematic review and economic evaluation. Health Technol Assess.

[7] Williams C, Brunskill S, Altman D, Briggs A, Campbell H, Clarke M, Glanville J, Gray A, Harris A, Johnston K, Lodge M (2006). Cost-effectiveness of using prognostic information to select women with breast cancer for adjuvant systemic therapy. Health Technol Assess.

[8] Stoehlmacher J, Park DJ, Zhang W, Yang D, Groshen S, Zahedy S, Lenz HJ (2004). A multivariate analysis of genomic polymorphisms: prediction of clinical outcome to 5-FU/oxaliplatin combination chemotherapy in refractory colorectal cancer. Br J Cancer.

[9] Ekhart C, Rodenhuis S, Smits PH, Beijnen JH, Huitema AD (2009). An overview of the relations between polymorphisms in drug metabolising enzymes and drug transporters and survival after cancer drug treatment. Cancer Treat Rev.

[10] Afsar NA, Haenisch S, Mateen A, Usman A, Ufer M, Ahmed KZ, Ahmad HR, Cascorbi I (2010). Genotype frequencies of selected drug metabolizing enzymes and ABC drug transporters among breast cancer patients on FAC chemotherapy. Basic Clin Pharmacol Toxicol.

[11] Tian C, Ambrosone CB, Darcy KM, Krivak TC, Armstrong DK, Bookman MA, Davis W, Zhao H, Moysich K, Gallion H, DeLoia JA (2012). Common variants in ABCB1, ABCC2 and ABCG2 genes and clinical outcomes among women with advanced stage ovarian cancer treated with platinum and taxane-based chemotherapy: a Gynecologic Oncology Group study. Gynecol Oncol.

[12] Vulsteke C, Pfeil AM, Schwenkglenks M, Pettengell R, Szucs TD, Lambrechts D, Peeters M, van Dam P, Dieudonné AS, Hatse S, Neven P, Paridaens R, Wildiers H (2014). Impact of genetic variability and treatment-related factors on outcome in early breast cancer patients receiving (neo-) adjuvant chemotherapy with 5-fluorouracil, epirubicin and cyclophosphamide, and docetaxel. Breast Cancer Res Treat.

[13] Hamidovic A, Hahn K, Kolesar J (2010). Clinical significance of ABCB1 genotyping in oncology. J Oncol Pharm Pract.

[14] Sharom FJ, You G, Morris ME (2007). Multidrug resistance protein: P-glycoprotein. Drug Transporters: Molecular Characterization and Role in Drug Disposition.

[15] Suzuki H, Sugiyama Y (2002). Single nucleotide polymorphisms in multidrug resistance associated protein 2 (MRP2/ABCC2): its impact on drug disposition. Adv Drug Deliv Rev.

[16] Han JY, Lim HS, Yoo YK, Shin ES, Park YH, Lee SY, Lee JE, Lee DH, Kim HT, Lee JS (2007). Associations of ABCB1, ABCC2, and ABCG2 polymorphisms with irinotecan-pharmacokinetics and clinical outcome in patients with advanced non-small cell lung cancer. Cancer.

[17] Nies AT, Rius M, Keppler D, You G, Morris ME (2007). Multidrug resistance proteins of the ABCC subfamily. Drug Transporters: Molecular Characterization and Role in Drug Disposition.

[18] Quintela-Fandino M, Hitt R, Medina PP, Gamarra S, Manso L, Cortes-Funes H, Sanchez-Cespedes M (2006). DNA-repair gene polymorphisms predict favorable clinical outcome among patients with advanced squamous cell carcinoma of the head and neck treated with cisplatin-based induction chemotherapy. J Clin Oncol.

[19] Bewick MA, Lafrenie RM, Conlon MS (2011). Nucleotide excision repair polymorphisms and survival outcome for patients with metastatic breast cancer. J Cancer Res Clin Oncol.

[20] Wu KG, He XF, Li YH, Xie WB, Huang X (2014). Association between the XPD/ERCC Lys751Gln polymorphism and risk of cancer: evidence from 224 case-control studies. Tumour Biol.

[21] Tengström M, Mannermaa A, Kosma VM, Soini Y, Hirvonen A, Kataja V (2014). MnSOD rs4880 and XPD rs13181 polymorphisms predict the survival of breast cancer patients treated with adjuvant tamoxifen. Acta Oncol.

[22] Aysola K, Desai A, Welch C, Xu J, Qin Y, Reddy V, Matthews R, Owens C, Okoli J, Beech DJ, Piyathilake CJ, Reddy SP, Rao VN (2013). Triple Negative Breast Cancer – An Overview. Hereditary Genet.

[23] Le Du F, Eckhardt BL, Lim B, Litton JK, Moulder S, Meric-Bernstam F, Gonzalez-Angulo AM, Ueno NT (2015). Is the future of personalized therapy in triple-negative breast cancer based on molecular subtype?. Oncotarget.

[24] Sharma S, Barry M, Gallagher DJ, Kell M, Sacchini V (2015). An overview of triple negative breast cancer for surgical oncologists. Surg Oncol.

[25] Arslan C, Dizdar O, Altundag K (2009). Pharmacotherapy of triple-negative breast cancer. Expert Opin Pharmacother.

[26] Kim JH, Kim SR, Song IS, Shin HJ, Kim HS, Lee JH, Ko SG, Shin YC (2011). Different transport activity of human triallelic MDR1 893Ala/Ser/Thr variant and its association with herb extracts. Phytother Res.

[27] Simon N, Marsot A, Villard E, Choquet S, Khe HX, Zahr N, Lechat P, Leblond V, Hulot JS (2013). Impact of ABCC2 polymorphisms on high-dose methotrexate pharmacokinetics in patients with lymphoid malignancy. Pharmacogenomics J.

[28] Benhamou S, Sarasin A (2002). ERCC2/XPD gene polymorphisms and cancer risk. Mutagenesis.

[29] Lunn RM, Helzlsouer KJ, Parshad R, Umbach DM, Harris EL, Sanford KK, Bell DA (2000). XPD polymorphisms: effects on DNA repair proficiency. Carcinogenesis.

[30] Chew HK, Doroshow JH, Frankel P, Margolin KA, Somlo G, Lenz HJ, Gordon M, Zhang W, Yang D, Russell C, Spicer D, Synold T, Bayer R, Hantel A, Stiff PJ, Tetef ML, Gandara DR, Albain KS (2009). Phase II studies of gemcitabine and cisplatin in heavily and minimally pretreated metastatic breast cancer. J Clin Oncol.

[31] da Silva Nogueira J, de Lima Marson FA, Sílvia Bertuzzo C (2012). Thymidylate synthase gene (TYMS) polymorphisms in sporadic and hereditary breast cancer. BMC Res Notes.

[32] Jamroziak K, Balcerczak E, Młynarski W, Mirowski M, Robak T (2002). Distribution of allelic variants of functional C3435T polymorphism of drug transporter MDR1 gene in a sample of Polish population. Pol J Pharmacol.

[33] Jerónimo C, Varzim G, Henrique R, Oliveira J, Bento MJ, Silva C, Lopes C, Sidransky D (2002). I105V polymorphism and promoter methylation of the GSTP1 gene in prostate adenocarcinoma. Cancer Epidemiol Biomarkers Prev.

[34] Khrunin AV, Moisseev A, Gorbunova V, Limborska S (2010). Genetic polymorphisms and the efficacy and toxicity of cisplatin-based chemotherapy in ovarian cancer patients. Pharmacogenomics J.

[35] Mammen JS, Pittman GS, Li Y, Abou-Zahr F, Bejjani BA, Bell DA, Strickland PT, Sutter TR (2003). Single amino acid mutations, but not common polymorphisms, decrease the activity of CYP1B1 against (−)benzo[a]pyrene-7R-trans-7,8-dihydrodiol. Carcinogenesis.

[36] Mitra AK, Singh N, Garg VK, Chaturvedi R, Sharma M, Rath SK (2009). Statistically significant association of the single nucleotide polymorphism (SNP) rs13181 (ERCC2) with predisposition to Squamous Cell Carcinomas of the Head and Neck (SCCHN) and Breast cancer in the north Indian population. J Exp Clin Cancer Res.

[37] Namazi S, Kojuri J, Khalili A, Azarpira N (2012). The impact of genetic polymorphisms of P2Y12, CYP3A5 and CYP2C19 on clopidogrel response variability in Iranian patients. Biochem Pharmacol.

[38] Przybylowska-Sygut K, Stanczyk M, Kusinska R, Kordek R, Majsterek I (2013). Association of the Arg194Trp and the Arg399Gln polymorphisms of the XRCC1 gene with risk occurrence and the response to adjuvant therapy among Polish women with breast cancer. Clin Breast Cancer.

[39] Qu J, Zhou BT, Yin JY, Xu XJ, Zhao YC, Lei GH, Tang Q, Zhou HH, Liu ZQ (2012). ABCC2 polymorphisms and haplotype are associated with drug resistance in Chinese epileptic patients. CNS Neurosci Ther.

[40] Rabachini T, Trottier H, Franco EL, Villa LL (2010). Validation of dot blot hybridization and denaturing high performance liquid chromatography as reliable methods for TP53 codon 72 genotyping in molecular epidemiologic studies. BMC Genet.

[41] Tseden-Ish M, Choi YD, Cho HJ, Ban HJ, Oh IJ, Kim KS, Song SY, Na KJ, Ahn SJ, Choi S, Kim YC (2012). Disease-free survival of patients after surgical resection of non-small cell lung carcinoma and correlation with excision repair cross-complementation group 1 expression and genotype. Respirology.

[42] Wilson MH, Grant PJ, Hardie LJ, Wild CP (2000). Glutathione S-transferase M1 null genotype is associated with a decreased risk of myocardial infarction. FASEB J.

